# Increased concentrations of soluble B7-H3 and interleukin 36 in bronchoalveolar lavage fluid of Children with *Mycoplasma pneumoniae* pneumonia

**DOI:** 10.1186/s12879-016-1555-6

**Published:** 2016-05-17

**Authors:** Zhengrong Chen, Xin Zhao, Xinxing Zhang, Guangbo Zhang, Huiming Sun, Wujun Jiang, Yuqing Wang, Canhong Zhu, Wei Ji, Yongdong Yan

**Affiliations:** Department of Respiratory Disease, Children’s Hospital of Soochow University, Soochow University, Suzhou, China; General surgery department, The First Affiliated Hospital of Soochow University, Soochow University, Suzhou, China; Clinical Immunology Laboratory, The First Affiliated Hospital of Soochow University, Soochow University, Suzhou, China

**Keywords:** *Mycoplasma pneumoniae* pneumonia, B7-H3, Interleukin 36, Children, Bronchoalveolar lavage fluid

## Abstract

**Background:**

The purpose of this study is to explore the correlations of interleukin 36 (IL-36) and Soluble B7-H3 (sB7-H3) levels in bronchoalveolar lavage fluid (BALF) with clinical characteristics and laboratory findings.

**Methods:**

A total of 35 children with *M. pneumnoiae* pneumonia (MPP) and 15 control subjects were enrolled. BALF concentrations of sB7-H3 and IL-36 were detected using enzyme-linked immunosorbent assays and clinical profiles of children with MPP were obtained.

**Results:**

Children with MPP had significantly higher levels of sB7-H3 and IL-36 compared to control subjects (both *P* < 0.05). Meanwhile, children with pleural effusion had significantly higher levels of sB7-H3 and IL-36 compared to children without pleural effusion (both *P* < 0.05). BALF concentration of sB7-H3 was strongly associated with concentration of IL-36 (*r* = 0.796, *P* < 0.0001) and sB7-H3 was correlated with duration of fever (*r* = 0.427, *P* = 0.11) and length of stay (*r* = 0.345, *P* = 0.043). Both concentrations of sB7-H3 and IL-36 were significantly decreased in convalescent phase after treatment (both *P* < 0.05).

**Conclusion:**

Both soluble B7-H3 and IL-36 may play an important role in pathogenesis of *M. pneumoniae* infection and sB7-H3 could be useful as a prognostic predictor or biomarker of MPP.

**Electronic supplementary material:**

The online version of this article (doi:10.1186/s12879-016-1555-6) contains supplementary material, which is available to authorized users.

## Background

Atypical pneumonia caused by *Mycoplasma pneumoniae* (*M. pneumoniae*) is a leading cause of mortality among the pediatric age group. *M. pneumoniae* was found in approximately 40 % of children infected with community acquired penumonia (CAP), among which 18 % of the patients required hospitalization [1]. In the past, *M. pneumoniae* infections were considered to occur mainly in older children, adolescents, and young adults. However, our recent studies have shown that *M. pneumoniae* has become an important cause of lower respiratory tract infection (LRTI) [2] or bronchiolitis [3] in infant patients using combined molecular and serologic methods.

*M. pneumoniae* infection can develop into a severe life-threatening disease such as acute respiratory distress syndrome, necrotizing pneumonitis, and fulminant pneumonia [4–6], although *M. pneumoniae* infection is usually a self-limited disease. Both pathogen invasion and host immune response play roles in severe *M. pneumoniae* infection. We have reported that increased levels of soluble B7-H3 (sB7-H3) and tumor necrosis factor-α (TNF-α) in peripheral blood may play an important role in immunopathogenesis of *M. pneumoniae* pneumonia (MPP) [7]. In vitro, *M. pneumoniae* could induce interleukin 1β expression and secretion in human monocytic U937 cell lines [8]. Interleukin 36 (IL-36), another member of the IL-1 gene family, including 3 agonistic cytokines, namely IL-36α, IL-36β, and IL-36γ, plays a major role in human psoriasis [9] as well as regulating airway inflammation [10] through stimulation on dendritic cells and CD4+ T cells [11, 12]. IL-36α can induce neutrophil influx and is also associated with increased mRNA expression of neutrophil-specific chemokines CXCL1 and CXCL2 in the lungs of C57BL/6 [13]. Meanwhile, IL-36 cytokines have a significant effect on the development of Th1 responses [14] which have a potential role in MPP [15], furthermore co-stimulatory molecule B7-H3 plays a vital role in T cells differentiation such as Th1 cells [16]. Thus, we presume that B7-H3 might be associated with IL-36 in patients with MPP.

So far, there is no study that reported the expression of IL-36 and sB7-H3 in bronchoalveolar lavage fluid (BALF) of children with MPP. The purpose of this study is to explore the associations between levels of IL-36 and sB7-H3 in BALF and clinical characteristics and laboratory findings.

## Methods

### Study design

From Janurary 2014 to December 2014, cases with MPP confirmed by both positive enzyme-linked immunosorbent assay (ELISA) and polymerase chain reaction (PCR) were enrolled. All cases were from 1 month to 14 years old and had fever, cough, tachypnea, chest retractions, abnormal auscultatory findings and radiologic evidence of CAP. Cases were not included if they had chronic lung disease, immunodeficiency, bronchopulmonary malformation, or co-infection. Fiberoptic bronchoscopy was applied if routine treatment of MPP (macrolide > 7 days and methylprednisolone > 2 days) was ineffective and patients still have a prolonged fever and radiological deterioration. Finally, BALF samples from 35 children diagnosed with MPP were collected. Radiograph of all patients were conducted 5–7 days after the first bronchoalveolar lavage and second bronchoalveolar lavage was performed if there were no definitive changes between radiographs. Meanwhile, 15 BALF samples of children with foreign body in bronchus were collected as control group. This study was approved by the Institutional Human Ethical Committee of Children’s Hospital of Soochow University and the methods were carried out in accordance with the approved guidelines. A written consent was obtained from all the guardians who participated in this study.

### Patient evaluation

The pediatricians completed a questionnaire regarding the demographic and clinical data on hospital admission and discharge. Chest radiographies of all cases were reviewed by the radiologists. BALF samples were collected from all of the enrolled children to detect common etiology using direct immunofluorescence assay (DFA) and PCRs. All the laboratory data was reviewed including blood rutine test, C-reactive protein concentration, immunoglobulin, subpopulation of T and B lymphocytes. sB7-H3 and IL-36 concentrations in BALF were detected using ELISA. Of all the cases, convalescent BALFs in 25 children were collected.

### Serology of *M. pneumoniae*

According to the manufacturer’s instructions, the specific antibodies against *M. pneumoniae* (IgG and IgM) were detected in serum samples of children including acute phase (upon admission) and convalescent phase (upon discharge) using a commercial ELISA kit (Serion ELISA classic *M. pneumoniae* IgG/IgM, Institute Virion/Serion, Würzburg, Germany). Acute *M. pneumoniae* infection was defined as either a single positive serum IgM titer (cut-off 13 U/mL) or a 4-fold increase in the IgG titer of convalescent serum.

### BALF collection and realtime PCR for *M. pneumoniae* detection

The procedure of BALF collection using fiber optic bronchoscopy was described previously [17]. First BALF samples were used for *M. pneumoniae* DNA, IL-36 and sB7-H3 detection and other BALF samples were used for cell count after centrifugation at 200 × g for 10 min at 4 °C. *M. pneumoniae* DNA was detected using a real-time PCR commercial kit (Daan Gene Co. Ltd, Guangzhou, China) as described previously [17]. In short, one sample of BALF was shaken for 30 s and centrifuged at 15,000 g for 5 min and the sediment was collected for DNA extraction from a 400-μl sample in accordance with the manufacturer’s instructions. PCR amplication was conducted using commercial primers and probes. Quantification curves were plotted using several concentrations of standard control samples.

### Multi-pathogen detection

Other nine viruses were detected using direct immunofluorescence assay or PCRs. Seven common viruses including respiratory syncytial virus, influenza virus types A and B, parainfluenza virus types 1–3, and adenovirus were detected using direct immunofluorescence assay while human metapneumovirus and human bocavirus were detected using PCRs as described previously [18].

### Examination of sB7-H3 and IL-36 in BALF

Collected BALF samples were preserved at −80 °C for subsequent assays after routine centrifugation. sB7-H3 ELISA kits were produced by our laboratory as previously described [19] and IL-36 ELISA kits were purchased from R&D company. All procedures were conducted according to the manufacturer’s instructions.

### Data analysis

The Chi-square test or Fisher’s exact test were applied if the data was numeral and the Student *t*-test or Mann–Whitney *U*-test were applied if the data distribution was non-normal. The Pearson or Spearman correlation test was used to assess correlations. A two-sided *p*-value of < 0.05 was considered statistically significant. All analyses were performed using SPSS for Windows, version 17.0 software (SPSS Inc., Chicago, IL, USA).

## Results

### Demographic and clinical data of children with MPP

The demographic data, clinical characteristics and laboratory findings of children with MPP are shown in Table 1. The average age of control subjects was 4.4 ± 2.4 (year), and the male percentage was 60 % (9/15). No statistical significance in age and gender was found between children with MPP and control subjects.

**Table 1:** Demographic and clinical data of children with MPP

Parameters	Childen with MPP
Age (mean ± SD, year)	5.5 ± 2.4
Male (n, %)	19 (54.3)
Duration of fever, (25th–75th percentile, d)	15 (13–18)
Length of stay, (25th–75th percentile, d)	10 (7–13)
White blood cell counts (mean ± SD, ×10^9^/L)	9.6 ± 4.596
Neutrophils proportion (mean ± SD, %)	70.1 ± 13.8
C-reactive protein (25th–75th percentile, mg/L)	25.4 (10.0–62.2)
ALT increase (n, %)	8 (22.9)
LDH (mean ± SD, U/L)	499.0 ± 171.8
CKMB (25th–75th percentile, U/L)	17.5 (15–24.4)
IgG (mean ± SD, g/L)	9.8 ± 4.2
IgA (25th–75th percentile, g/L)	1.4 ± 0.9
IgM (mean ± SD, g/L)	2.3 ± 1.2
CD3 + T cells (mean ± SD, %)	67.1 ± 10.9
CD3 + CD4+ T cells (mean ± SD, %)	36.6 ± 9.7
CD3-CD8+ T cells (mean ± SD, %)	26.2 ± 6.1
CD3-CD19+ B cells (mean ± SD, %)	19.2 ± 8.1
CD3-CD (16 + 56+) NK cells (mean ± SD, %)	12.0 ± 8.1
CD19 + CD23+ B cells (mean ± SD, %)	8.9 ± 4.7
Cytology of BALF	
Neutrophils (%)	65.6
Lymphocytes (%)	7.2
Phagocytes (%)	26.4
Radiologic evaluation (n, %)	
Lobar or segmental opacity	33 (94.3)
Opacity with pleural effusion	12 (34.3)
Opacity with pulmonary atelectasis	3 (8.6)
Macrolide medication (n, %)	35 (100)
Methylprednisolone (n, %)	35 (100)

*MPP* Mycoplasma pneumoniae pneumonia, *ALT* alanine transarninase, *LDH* L-lactate dehydrogenase, *CKMB* MB isoenzyme of creatine kinase, *Ig* immunoglobulin, *CD* cluster of differentiation

### BALF concentrations of sB7-H3 and IL-36 in children with MPP and control subjects

**Fig. 1:**
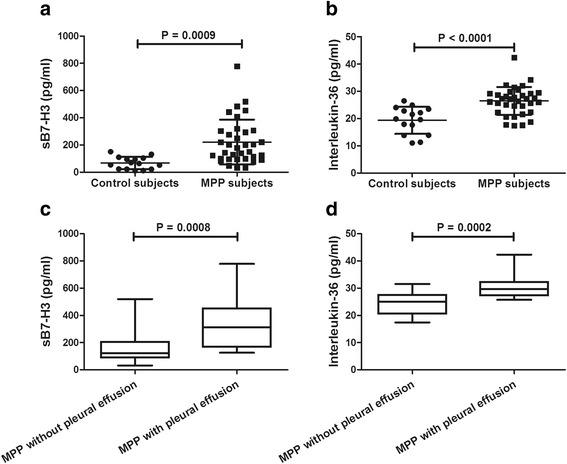
Comparison of BALF concentration of sB7-H3 (**a**) and IL-36 (**b**) between children with MPP and control subjects as well as comparison of sB7-H3 (**c**) and IL-36 (**d**) between children with and without pleural effusion

As shown in Fig. 1, children with MPP had significantly higher levels of sB7-H3 (221.3 ± 164.5 vs. 67.6 ± 45.0, pg/ml; *P* = 0.0009) and IL-36 (26.5 ± 5.2 vs. 19.4 ± 5.0, pg/ml; *P* < 0.0001) compared to control subjects. Among all *M. pneumoniae* infected cases, children with pleural effusion had significantly higher levels of sB7-H3 (342.6 ± 186.7 vs. 158.0 ± 110.3, pg/ml; *P* = 0.0008) and IL-36 (30.6 ± 4.5 vs. 24.3 ± 4.1, pg/ml; *P* = 0.002) compared to children without pleural effusion.

### Correlation between sB7-H3, IL-36 and clinical profiles in children with MPP

**Fig. 2:**
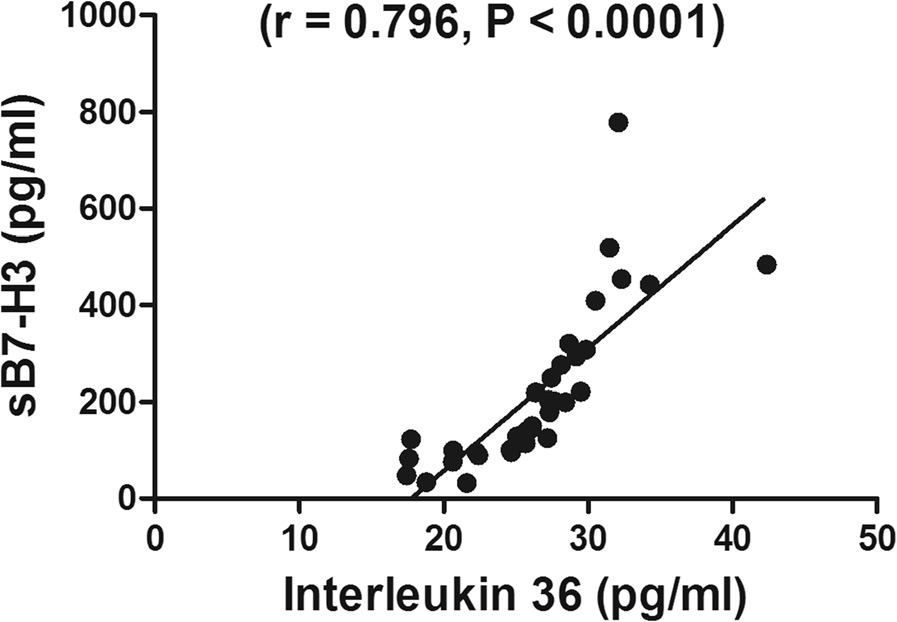
Correlation between BALF concentration of sB7-H3 and IL-36

To our interest, BALF concentration of sB7-H3 was strongly associated with concentration of IL-36 as shown in Fig. 2. As shown in Table 2, BALF concentration of sB7-H3 was correlated with duration of fever (*r* = 0.427, *P* = 0.011) and length of stay (*r* = 0.345, *P* = 0.043), however no significant correlation was found between IL-36 and clinical parameters.

**Table 2:** Correlation between sB7-H3, IL-36 and clinical profiles in children with MPP

Parameters	IL-36		sB7-H3	
	r	P	r	P
Duration of fever (d)	0.144	0.409	0.427	0.011
Length of stay (d)	0.162	0.352	0.345	0.043
Wite blood cell counts (×10^9^/L)	−0.020	0.910	−0.143	0.411
Neutrophils percentage (%)	0.000	0.999	0.081	0.643
C-reactive protein (mg/L)	0.100	0.566	0.113	0.518
ALT increase (%)	−0.169	0.332	−0.185	0.187
LDH (U/L)	0.039	0.825	0.233	0.178
CKMB (U/L)	0.006	0.971	−0.095	0.587
IgG (g/L)	0.203	0.241	0.027	0.879
IgA (g/L)	−0.008	0.963	−0.117	0.505
IgM (g/L)	0.080	0.649	0.068	0.696
CD3 + T cells (%)	−0.092	0.597	−0.184	0.289
CD3 + CD4+ T cells (%)	−0.050	0.774	0.004	0.982
CD3-CD8+ T cells (%)	−0.023	0.897	−0.141	0.421
CD3-CD19+ B cells (%)	−0.042	0.809	−0.041	0.815
CD3-CD(16 + 56+)NK cells (%)	0.167	0.337	0.227	0.190
CD19 + CD23+ B cells (%)	−0.163	0.349	−0.173	0.321
Cytology of BALF				
Neutrophils (%)	−0.221	0.201	−0.333	0.050
Lymphocytes (%)	0.089	0.610	0.048	0.786
Phagocytes (%)	0.169	0.331	0.294	0.087

*MPP* Mycoplasma pneumoniae pneumonia, *ALT* alanine transarninase, *LDH* L-lactate dehydrogenase, *CKMB* MB isoenzyme of creatine kinase; Ig: immunoglobulin, *CD* cluster of differentiation. *P* < 0.05 was considered statistically significant

### BALF concentrations of sB7-H3 and IL-36 before and after treatment

**Fig. 3:**
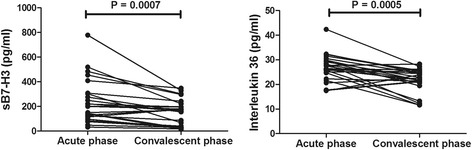
BALF concentrations of sB7-H3 and IL-36 in children with MPP before and after treatment

All children were treated with Azithromycin (10 mg/kg.d) and Methylprednisolone (1-2 mg/kg.d) and no severe complications (bronchiolitis obliterans, bronchiectasis) were found in any case within 6 months follow-up. Convalescent BALF samples were obtained from 25 children with MPP. Both concentrations of sB7-H3 and IL-36 significantly decreased in convalescent phase as shown in Fig. 3.

## Discussion

This study describes the expression of sB7-H3 and IL-36 in BALF samples of children with MPP and explores the correlations with clinical profiles. Our study shows that both BALF concentrations of sB7-H3 and IL-36 in children with MPP were significantly increased compared to control subjects and strong correlation was found between sB7-H3 and IL-36. In addition, children with pleural effusion had significantly higher concentrations of sB7-H3 and IL-36. Furthermore, BALF concentration of sB7-H3 was associated to duration of fever and hospital stay. We presume that sB7-H3 may be an important prognostic indicator to evaluate the disease severity.

Generally speaking, host immune response caused by *M. pneumoniae* is a double-edged sword. On one hand, host immune response developed by *M. pneumoniae* infection plays a role in protection from disease deterioration. On the other hand, it could lead to excessive inflammatory response in some cases and consequently developed into severe *M. pneumoniae* infection especially in older children. A wide range of cytokines and chemokines were generated in the respiratory tracts infected by *M. pneumoniae*, including TNF-α, IFN-γ, IL-6, IL-8, IL-17, IL-18 [20–22] and then caused inflammatory infiltration by neutrophils and lymphocytes [23]. In present study, pleural effusion was a sign of excessive inflammation indirectly induced by *M. pneumoniae* infection because no evidence of *M. pneumoniae* in pleural effusions from 3 childen was found using PCR method (data not shown).

B7-H3, a co-stimulatory molecule, plays an important role in the regulation of both innate and Ag-specific T cell-mediated immune responses and inflammation [16, 24, 25]. In our previous study, B7-H3 could be induced by lipoproteins and functioned as a co-stimulator of innate immunity by augmenting pro-inflammatory cytokine (TNF-α, IL-6) release from bacterial cell wall product-stimulated monocytes/macrophages [25]. In a murine model of pneumococcal meningitis, B7-H3 could augment pro-inflammatory cytokine and chemokine production, upregulate NF-κB p65 and MAPK p38 phosphorylation, and enhance the nuclear transactivation of NF-κB p65 through TLR2-dependent mechanism [26]. Lipoproteins derived from *M. pneumoniae* could induce infiltration of leukocyte cells and production of chemokines and cytokines in BALF [27]. In a word, B7-H3 takes part in inflammatory pathogenesis of MPP and is a good prognostic indicator.

IL-36, a cytokine of IL-1 family member, could express both in epithelia and in immune cells [28]. A recent study [14] demonstrated that IL-36 stimulates the activation of naive CD4+ T cell proliferation and IL-2 production. Meanwhile, IL-36 signaling was also critical for Th1-protective immune responses in an experimental model of Bacillus Calmette-Guerin infection [14]. Consequently, IL-36 may act as a signal to activate cells of the innate and adaptive immunity such as DCs and naive CD4+ T cells to stimulate host responses against pathogens. This is the first time the high expression of IL-36 in children with MPP and the strong correlation with sB7-H3 were reported. All things considered, we presume that both B7-H3 and IL-36 are crucial in immunopathogenesis of *M. pneumoniae* infection and further studies should be done to elucidate the specific mechanism.

## Conclusions

To summarize, these data support that sB7-H3 and IL-36 may play important roles in inflammatory pathogenesis of *M. pneumoniae* infection. sB7-H3 could be a useful prognostic predictor or biomarker of MPP.

## Ethics and consent to participate

This study was approved by the Institutional Human Ethical Committee of Children’s Hospital of Soochow University and the methods were carried out in accordance with the approved guidelines. A written consent was obtained from all the guardians who participated in this study.

## Consent to publish

Not applicable.

## Availability of data and materials

The datasets supporting the conclusions of this article are included within the article and its Additional file 1.

## Additional file

Additional file 1: Supporting data. (XLS 58 kb)

## Abbreviations

BALF: bronchoalveolar lavage fluid
CAP: community acquired penumonia
ELISA: enzyme linked immunosorbent assay
IL-36: interleukin 36
LRTI: lower respiratory tract infection
M. pneumoniae: *Mycoplasma pneumoniae*
MPP: *Mycoplasma pneumoniae* pneumnia
PCR: polymerase chain reaction
sB7-H3: Soluble B7-H3
TNF-α: tumor necrosis factor-α
